# Magnetic Bragg dip and Bragg edge in neutron transmission spectra of typical spin superstructures

**DOI:** 10.1038/s41598-017-15850-3

**Published:** 2017-11-14

**Authors:** Hiroaki Mamiya, Yojiro Oba, Noriki Terada, Norimichi Watanabe, Kosuke Hiroi, Takenao Shinohara, Kenichi Oikawa

**Affiliations:** 1 National Institute for Materials Science, Tsukuba, 305–0047 Japan; 2 Japan Atomic Energy Agency, Tokai, 319–1195 Japan; 3 Present Address: Kanagawa University, Yokohama, 221-8686 Japan

## Abstract

Neutron diffractometry has been a critical tool for clarifying spin structures. In contrast, little attention has been paid to neutron transmission spectroscopy, even though they are different types of the same phenomenon. Soon, it will be possible to measure the wavelength dependence of transmissions easily using accelerator-driven neutron facilities. Therefore, we have started studying the potential of spectroscopy in magnetism, and in this paper, we report the first observation of a magnetic Bragg dip and Bragg edge in the neutron transmission spectra of a typical spin superstructure; clear antiferromagnetic Bragg dips and Bragg edges are found for a single crystal and powder of nickel oxide, respectively. The obtained results show that transmission spectroscopy is a promising tool for measurements under multi-extreme conditions and for the precise analyses of spin structures, not only in MW-class pulsed spallation source facilities but also in compact neutron source facilities.

## Introduction

Extreme conditions such as high pressure, high magnetic field, and low temperature are attractive frontiers in magnetism. Neutron diffractometry is a unique tool for clarifying spin arrangements directly. Therefore, different types of sample environment devices for neutron diffractometry have been investigated for experiments under high-field and/or high-pressure and low-temperature conditions^1–4^. However, it is not easy to design such devices, particularly for multi-extreme conditions with tight restrictions in the arrangement of the beam path, because the scattered beam is not on the same straight line as the incident beam. This difficulty is enhanced for single crystals as the directions of the scattered beam are highly restricted within specified directions^3,4^. Hence, alternative methods for diffractometry are necessary for exploring the frontiers; however, extremely few methods have been proposed over the past few decades.

**Figure 1:**
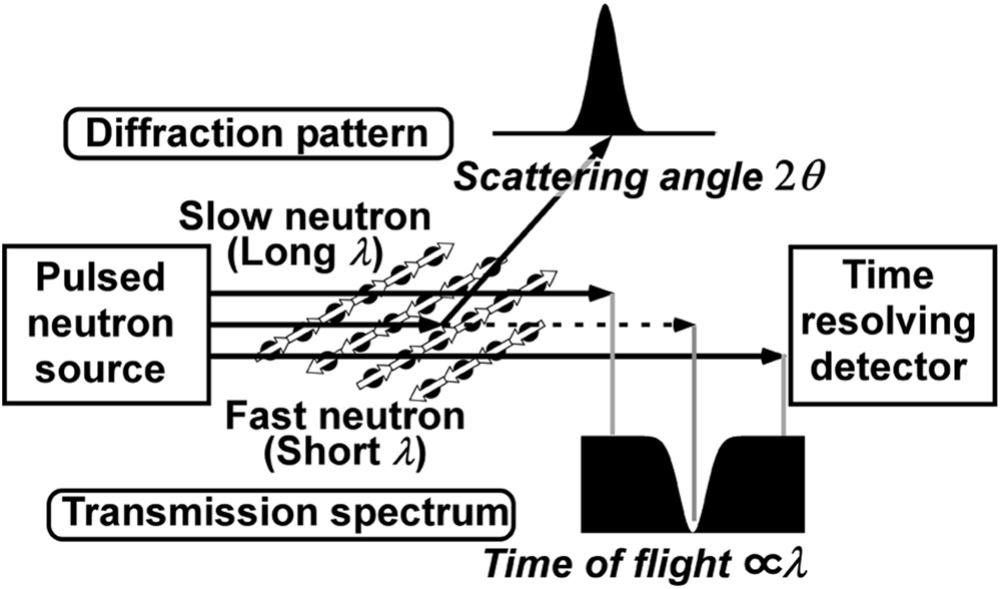
Schematic diagram of neutron transmission spectroscopy. Neutrons with velocity distributions, generated in a pulsed neutron source, are detected through a sample. A Bragg dip is observed in the time of flight spectrum if the neutrons with a specific wavelength *λ* are scattered by Bragg reflection because wavelength is inversely proportional to velocity.

In recent years, wavelength-dependent neutron transmission spectroscopy that uses new spallation neutron sources has provided a novel research field related to system engineering. Step-like or dip-like decreases, referred to as Bragg edges or Bragg dips, have been observed in neutron transmission spectra because Bragg reflections weaken the transmission at certain wavelengths in polycrystalline and single-crystalline materials, as illustrated in Fig. 1 ^5–8^. Based on this, we can derive crystallographic information related to the phase volume fraction, preferred orientation, or elastic strain on the transmission path from the transmission spectroscopy as well as from diffractometry. Therefore, considerable attention has been paid to nondestructive imaging for residual strains or texture evolution in structures such as automobile engines, gas turbine blades, and welding tubes by utilizing accelerator-based instruments equipped with highly penetrating pulsed neutron sources and time-resolved two-dimensional neutron detectors.

At this stage, we must note that such a neutron transmission spectrum can be detected on the same line of the incident beam; hence, the flexibility of design can be enhanced while optimizing the equipment and simultaneously realizing high-field, high-pressure, and low-temperature operation. In other words, the methods that use Bragg dips or Bragg edges expand the research opportunities in the area of extreme-condition magnetism. However, extremely few studies have been conducted on the variations in the transmission spectrum owing to the magnetic scattering from the spin superstructures. For this reason, we attempt to clarify the rich potential of spectroscopy in magnetism. In this paper, we report the first observation of a magnetic Bragg dip and Bragg edge in the neutron transmission spectra of a typical spin superstructure: a collinear antiferromagnetic superstructure of a {111} ferromagnetic sheet in a single crystal and powder of nickel oxide.

## Magnetic Bragg dip and edge in neutron transmission spectrum

The wavelength-dependent neutron transmission spectrum, *Tra*(*λ*), is represented by the Beer–Lambert–Bouguer law as $Tra(\lambda )=exp(-{\sigma }_{tot}\rho {t}_{h})$, where *σ*_tot_ is the neutron total cross section, *ρ* is the density, and *t*_h_ is the thickness of the sample^7^. For thermal neutrons, *σ*_tot_ is expressed as follows:

1 $${\sigma }_{tot}={\sigma }_{{\rm{coh}}}^{{\rm{ela}}}+{\sigma }_{{\rm{incoh}}}^{{\rm{ela}}}+{\sigma }_{{\rm{coh}}}^{{\rm{inela}}}+{\sigma }_{{\rm{incoh}}}^{{\rm{inela}}}+{\sigma }_{{\rm{abs}}},$$

where ${{\sigma }}_{\mathrm{coh}}^{\mathrm{ela}}$, ${{\sigma }}_{\mathrm{incoh}}^{\mathrm{ela}}$, ${{\sigma }}_{\mathrm{coh}}^{\mathrm{inela}}$, and ${{\sigma }}_{\mathrm{incoh}}^{\mathrm{inela}}$ represent elastic coherent, elastic incoherent, inelastic coherent, and inelastic incoherent scattering cross sections, respectively, and *σ*_abs_ denotes the absorption cross section. In this study, we focus on the first term because the dependences of the other terms on *λ* are generally monotonous or insignificant for thermal neutrons. According to the diffraction theory for the Bragg scattering of unpolarized neutrons from a periodic structure, the differential cross section, ${\rm{d}}{{\sigma }}_{\mathrm{coh}}^{\mathrm{ela}}/{\rm{d}}$Ω, can be described as

2 $$\frac{d{\sigma }_{{\rm{coh}}}^{{\rm{ela}}}}{{\rm{d}}{\Omega }}=N\frac{{(2\pi )}^{3}}{{v}_{{\rm{o}}}}{\sum }_{\tau }(|{F}_{N}({\boldsymbol{q}}){|}^{2}+|{F}_{M}({\boldsymbol{q}}){|}^{2})\delta ({\boldsymbol{q}}-{{\boldsymbol{Q}}}_{\tau }),$$

where *N* is the total number of unit cells, *v*_0_ is the unit cell volume, ***q*** is the unit scattering vector, ***Q***_τ_ represents the reciprocal lattice vectors of the periodic structure, and *F*_N_(***q***) and *F*_M_(***q***) are the crystal and magnetic structure factors, respectively^9^. Consequently, ${{\sigma }}_{\mathrm{coh}}^{\mathrm{ela}}$ is given by

3 $${\sigma }_{{\rm{coh}}}^{{\rm{ela}}}=N\frac{\,\pi {\lambda }^{2}}{{v}_{0}}{\sum }_{\tau }{{Q}_{\tau }}^{-1}({|{F}_{{\rm{N}}}({{\boldsymbol{Q}}}_{\tau })|}^{2}+{|{F}_{{\rm{M}}}({{\boldsymbol{Q}}}_{\tau })|}^{2})$$

For a periodic spin order in a single crystal, we can expect to observe countable dips, referred to as magnetic Bragg dips, in *Tra*(*λ*) at *λ* where the Laue condition is satisfied. A notable point is that all reflections can be detected in *Tra*(*λ*) as dips in a single spectrum, though the components of the magnetic moments parallel to ***q*** cannot contribute to such dips as they typically do. On the other hand, numerous dips must appear for polycrystalline powders. It is worthwhile to note that no reflections from the (hkl) plane can occur in the *λ* range longer than double the length of the interplanar spacing, *d*_hkl_. Consequently, we can observe a cut-off wavelength for attenuation, referred to as the magnetic Bragg edge, at 2*d*_hkl_, for periodic spin structures.

## Results

**Figure 2:**
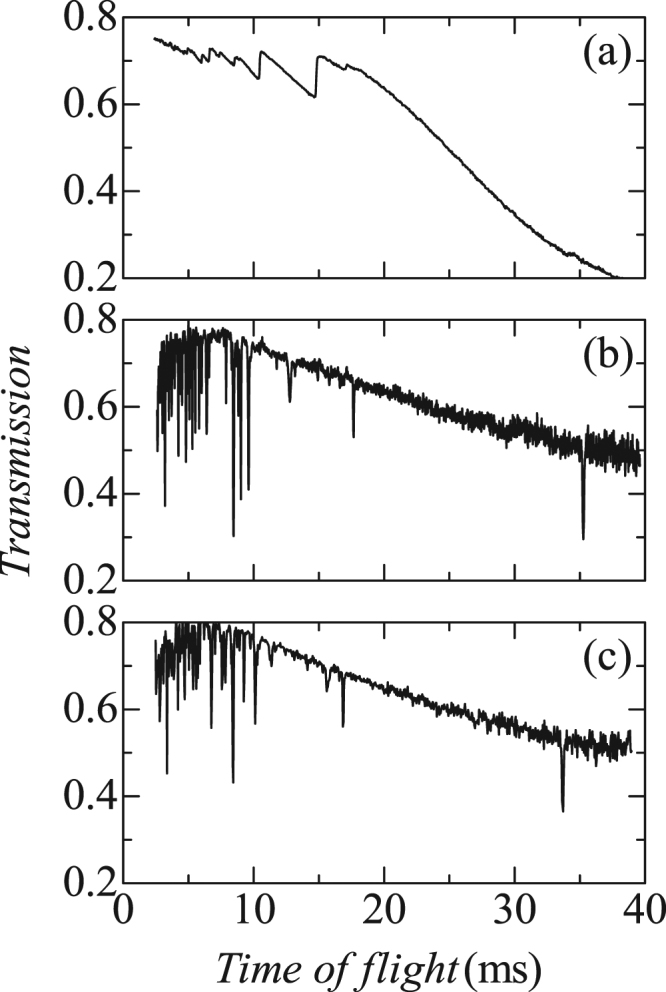
Neutron transmission of nickel oxide as a function of time of flight *t* for (**a**) the randomly packed powder, (**b**) the perpendicularly fixed single crystal, and (**c**) the purposely inclined single crystal.

**Figure 3:**
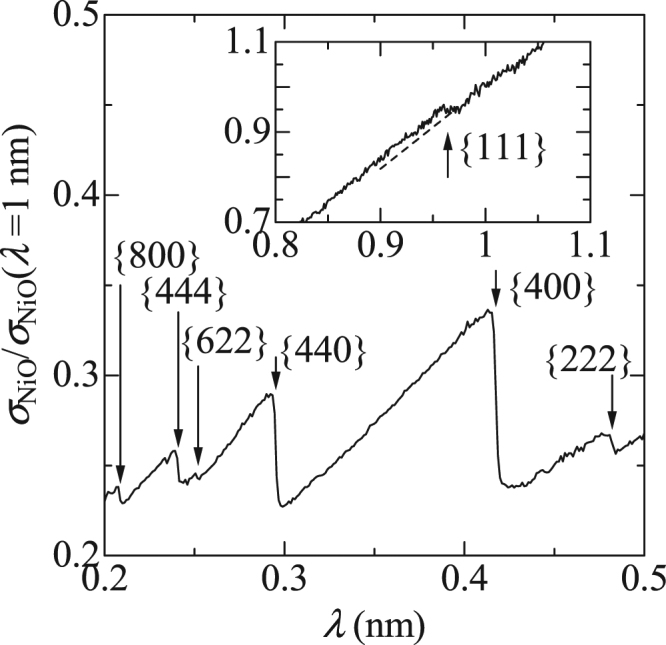
Wavelength-dependent total cross section, *σ*_tot_, for the randomly packed powder. The downward arrows indicate the values of 2*d*_hkl_ for the {hkl} atomic planes. Inset shows *σ*_tot_ at longer λ, where the upward arrow indicates the value of 2*d*_hkl_ for an antiferromagnetic periodic structure; the broken line is a visual guide.

**Figure 4:**
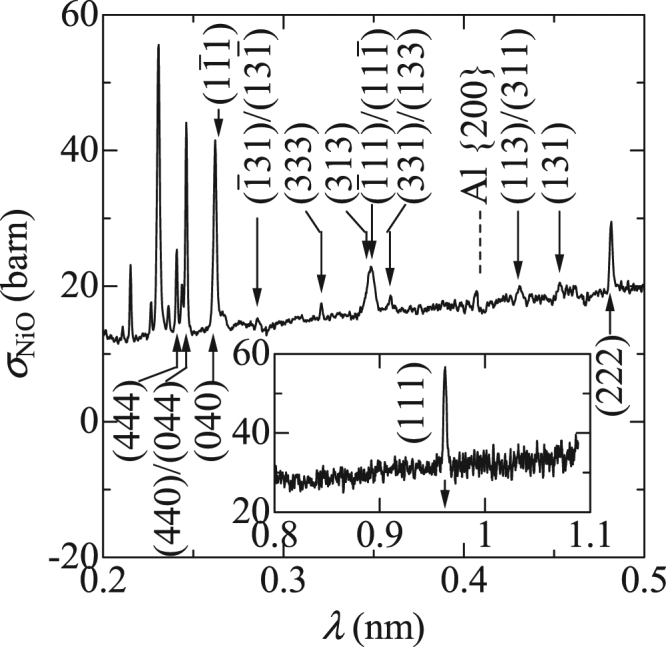
Wavelength-dependent total cross section, *σ*_tot_, for the perpendicularly fixed single crystal, where the peak positions calculated for nuclear and magnetic scatterings of the (hkl) planes are shown by the upward and downward arrows, respectively. Inset shows *σ*_tot_ at longer λ.

**Figure 5:**
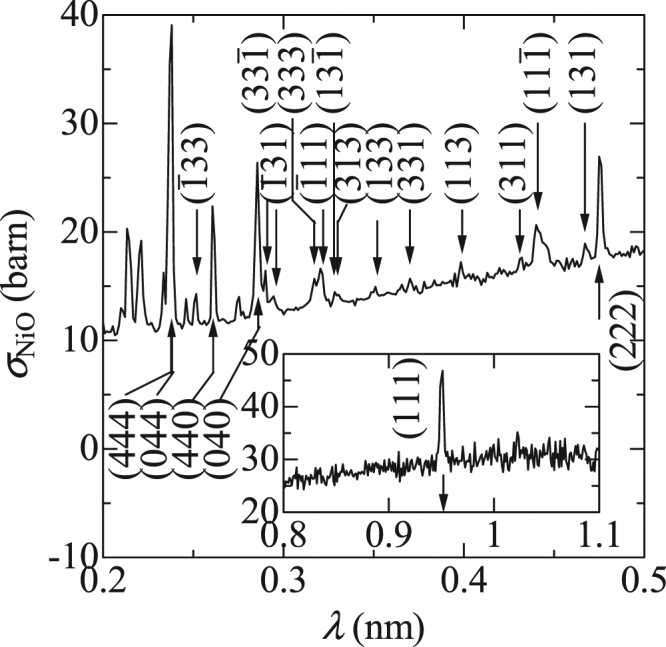
Wavelength-dependent total cross section, *σ*_tot_, for the purposely inclined single crystal, where the peak positions calculated for nuclear and magnetic scatterings of (hkl) planes are shown by the upward and downward arrows, respectively. Inset shows *σ*_tot_ at longer λ.

Figure 2 shows the neutron transmissions of a single crystal and powder of nickel oxide (NiO) as functions of the time of flight, *t*. It can be seen that the transmission of the powder, shown in (a), gradually decreases with several step-like increases, as the time of flight increases. On the other hand, we find frequent dips in the transmission spectra of the single crystal, as shown in (b) and (c). We can expect that the former steps and latter dips are related to the concerned Bragg edges and Bragg dips, respectively. The wavelength-dependent total cross sections, *σ*_tot_, estimated from the observed results are shown in Figs 3–5 to clarify the relationship further, where *σ*_tot_ for the powder is given as a relative ratio owing to the lack of information on the exact thickness.

In Fig. 3, several saw-tooth shapes can be observed in the spectrum of *σ*_tot_ for the NiO powder. We use a cubic unit cell, with double the period of the original chemical cell, for simultaneous analyses of the magnetic superstructure and crystalline structure, as performed in a previous study on neutron diffractometry^10^, where lattice constant *a* is 0.835 nm. The downward arrows in Fig. 3 indicate the values of 2*d*_hkl_ for the {hkl} atomic planes. Their positions coincide with the drop-off wavelengths in the spectrum. Hence, these can be attributed to the Bragg edges due to nuclear scattering. There is an extra step at *λ* = 0.96 nm, where the step height is almost 1%. This length corresponds to twice the period for a collinear antiferromagnetic superstructure of a {111} ferromagnetic sheet. For this reason, we can consider the observed step as a Bragg edge due to magnetic scattering. In a previous study^10^, it was reported that the intensity of the {111} magnetic reflection was almost comparable to that of the {222} nuclear reflection. As expected from this result, we are certain that the magnetic Bragg edge height observed in Fig. 3 is 0.01 and almost the same as that for the {222} nuclear reflection.

In Fig. 4, we can observe several peaks in the spectrum of *σ*_tot_ for the single NiO crystal where the (111) surface is set approximately perpendicular to the incident beam. According to the Laue condition, the Bragg reflections for a cubic crystal should occur at

4 $$\lambda =2{d}_{hkl}\cdot \,\sin \,\theta =2a\cdot (hH+kK+lL)/({h}^{2}+{k}^{2}+{l}^{2}),$$

where *θ* is the scattering angle and < *HKL* > is a unit vector parallel to the direction of the incident neutron beam. This formula shows that the three major reflections for (444), (440), and (040), which were reported previously^10^, should be observed at the same *λ* of $a/(2\sqrt{3})=0.241$ nm, if <*HKL*> is parallel to <111>. However, we find several split peaks around 0.24 nm in Fig. 4. Therefore, we fit the peak positions in the range between the (444) and (111) peaks based on the assumption that the (111) surface is not exactly perpendicular to the incident beam. The peak positions calculated for nuclear and magnetic scatterings are shown by the upward and downward arrows, respectively, where < *HKL* > is < 0.551 0.626 0.551 > (the tilted angle is 3.5°). All observed peaks can be assigned to not only nuclear but also magnetic scatterings. This is the first observation of the Bragg dip due to spin ordering in a single crystal. This single crystal contains multi-antiferromagnetic domains, as we can observe the $(\bar{1}11)$, $(1\bar{1}1)$, and $(11\bar{1})$ peaks in addition to the (111) peak. It is notable that all expected scatterings can be confirmed in the spectrum, even though the crystal–plane alignment is incomplete.

Figure 5 shows the spectrum of *σ*_tot_ for the single NiO crystal with the (111) surface purposely inclined further (almost 10° from the perpendicular.) The splitting appears to be enlarged. Hence, the peak positions are adjusted by varying the tilting angle. The peak positions calculated for the nuclear and magnetic scatterings in the range between the (444) and (111) peaks are shown by the upward and downward arrows, respectively, where < *HKL* > is < 0.565 0.685 0.460 > (the tilted angle is 9.2°). We were able to find all expected scatterings without losing them even if the inclination was increased further.

## Discussion

As shown in the previous section, we succeeded in observing a Bragg edge and Bragg dip due to magnetic scattering from spin superstructures in the transmission spectra of the powder and single crystal of NiO, respectively. These spectra and the previously observed diffraction patterns are different types of the same phenomenon. However, spectroscopy exhibits certain advantages and disadvantages compared with diffractometry, which are discussed below.

For single crystals, the following points can be considered:

- Designability: Most external fields such as magnetic, electric, or stress fields have axial natures; hence, their multiple application is fundamentally compatible with neither the wide-angle window nor the plural windows for neutrons. On the contrary, the neutron transmission spectrum can be detected using a pair of small windows on the same line as the incident beam (Fig. 1). Hence, the flexibility of design for sample environment devices under multi-extreme conditions can be improved significantly.
- Sample size: Smaller samples are preferably used in experiments performed under multi-extreme conditions^11^. In Fig. 2(b), the dip depth and the full width at half of the maximum of the (111) magnetic scattering for a sample of thickness 4 mm are approximately 40% and 0.4%, respectively. The resolution of the time of flight is 0.3–0.4% in a beam line 10 NOBORU obtained using a decoupled moderator^12^. Hence, the observed dip width is essentially attributed to the resolution. In other words, the dip becomes deeper if higher resolution instruments such as poisoned moderators^13^ are used. Based on this argument, the dip depth can be expected to be 10% or more, even for a thickness of 1 mm.
  In the experiment shown in Fig. 2(b), the incident neutron beam was weakened using smaller collimators because the count rate of the boron-coated gas electron multiplier (nGEM) detector was not sufficient. Consequently, a neutron beam with a flux density of 1 × 10^2^ n/s/mm^2^ was irradiated on a (111) surface with an area of 10 mm × 10 mm. However, it is possible to generate a thin neutron beam with a size of 1 mm × 1 mm and an incident flux of 1 × 10^6^ n/s/mm^2^ in recently developed pulsed spallation neutron sources using a MW-class high-power proton beam. In the recently developed scintillators that use cerium-doped LiCaAlF_6_ crystals, the typical decay time for the 5d-4f luminescence is 40 ns^14^; hence, it can count neutrons at rates of more than 1 × 10^6^ n/s accurately. In other words, the total count can be increased hundredfold even if the irradiated area is reduced to 1 mm × 1 mm. In summary, a size of 1 mm^3^ is practical for studying spin superstructures such as NiO (1.9 μ_B_^10^) because the above estimation shows that dips with depths deeper than 10% can be expected, with 10 times improvement in the signal-to-noise ratio.
- Workability: High accuracy of crystal orientation is typically required for experiments under multi-extreme conditions because scattered neutrons must pass through small windows of sample cells and be caught by the detectors behind them. It is not easy to load a small crystal with a size of 1 mm into sample cells and stabilize it in the desired direction. When the opening angle of the window or detector is a few degrees, the accuracy is better than that necessary for the alignment of inclination and/or rotation. On the contrary, transmitted neutrons are always on the same line as the incident beam, regardless of the direction of the crystal, as discussed above. Furthermore, the observed Bragg dips can be indexed even if the crystal is slightly tilted, as shown in Figs 3 and 4.
- Information: We cannot derive the directions of the scattering planes from the analyses of Bragg dips, in contrast to the single-crystal diffractometers with multi-axis goniometers and large-area detectors. At this stage, it must be noted that mechanically rotating stages and wide-angle windows are not common in experiments under multi-extreme conditions because they are unsuitable for satisfying the design constraints. In such experiments, the crystal is carefully set in a specific direction beforehand so that the diffraction spot can be observed through the windows. Consequently, with regard to the direction, the experimental results only show that the scattered planes are aligned as planned. In this context, we can infer that the information derived from spectroscopy is not poor in comparison to that obtained from single-crystal diffractometry, in the area of extreme-condition magnetism.
  Furthermore, it is not easy to continuously observe the diffraction spot in the small window when unexpected transitions of spin configuration occur. On the contrary, all dips caused by the reflections can be found in the spectrum even if the spin structure is changed completely. Thus, the measurements of Bragg dips are useful for studying the variations in the spin configuration under multi-extreme conditions.
  As discussed here, the transmission spectroscopy for the magnetic Bragg dips of single crystals have noticeable advantages while studying the magnetic structures under multi-extreme conditions. In contrast, we cannot fully utilize this type of advantage to study the spin structures under multi-extreme conditions in powder samples from the viewpoint of sample size. The depths of the Bragg edges are considerably shallower than that of the Bragg dips because only the grains whose scattering planes are stochastically arranged vertical to the incident beam contribute to the formation of edge jump. This can be observed in Fig. 2, although their thicknesses are not exactly the same. Consequently, it is difficult to decrease the effective thickness of the samples with atomic magnetic moments comparable to those of NiO (1.9 μ_B_.) A few exceptions are rare-earth compounds, such as SmMnO_3_^15^ and TbMnO_3_^16^, because their edge heights ${|{F}_{{\rm{M}}}({{\boldsymbol{Q}}}_{{\tau }})|}^{2}$ are proportional to the squares of the sizes of their magnetic moments (e.g., 10 μ_B_ for TbMnO_3_). The difficulty in reducing sample size is a major disadvantage of using transmission spectroscopy to study the magnetic structures of powder samples under multi-extreme conditions. We shall now consider other advantages of measuring the Bragg edges. Next, we note the viewpoints of resolution and sensitivity, even though we can principally derive the same information from the Bragg edges in the transmission spectrum as neutron powder diffractions.
- Resolution: Generally, the resolution of neutron powder diffraction using pulsed spallation sources is given by
  5 $$\Delta d/d\,={[{(\Delta t/t)}^{2}+{(\Delta L/L)}^{2}+{(\cot \theta {\Delta }\theta )}^{2}]}^{1/2},$$
  where *Δt* is a timing uncertainty due to the width of the initial pulse, *θ* and *Δθ* are the scattering angle and its uncertainty, respectively, *L* and *ΔL* are the total flight path length from the moderator to the detector via the sample and its uncertainty, respectively^17^. The third term in Eq. (5) is not applicable in transmission spectroscopy because the spectra of randomly oriented powders are independent of the incident directions of neutrons. In other words, it is unnecessary to pay attention to the incident beam divergence for high resolutions. Furthermore, *L* is just the distance between the moderator and detector in transmission spectroscopy; hence, *L* is independent of the scattering position in the sample. In other words, we do not need to consider sample size for high resolution. A more important point is the high flux of the transmitted beam in comparison with the flux of scattered neutrons. In conventional diffractometry, sufficient intensity of a signal is obtained by reducing the resolution because a narrower pulse width (*Δt*), longer flight path (*t*, *L*), thinner moderator (*Δt*, *ΔL*), and thinner detector (*ΔL*) have trade-off relationships with the higher incident flux at the sample and higher detection efficiency. On the contrary, the magnetic Bragg edge was detectable using a weak incident beam at a flux of 6 × 10^2^ n/s/mm^2^, as shown in Fig. 3. This result shows that we can optimize the neutron optical system for high resolution without considering the reduction in the incident beam in MW-class facilities whose incident beam flux is generally 1 × 10^6^ n/s/mm^2^.
- Sensitivity: Transmission spectroscopy provides an intrinsic disadvantage while measuring weak reflections. In our experiment, the detector counted a total of 4 × 10^4^ neutrons every 40 μs of the time of flight, around the (111) magnetic Bragg edge at *t* = 34 ms. Therefore, we can barely distinguish the existence of the edge with a height of 2% (800 neutrons) in the presence of statistical errors of $\sqrt{{4\times 10}^{4}}$ counts in the present spectroscopy (see Fig. 2). On the contrary, 800 scattered neutrons can be easily observed in statistical errors of $\sqrt{800}$ in general diffractometry. A simple solution to overcome this disadvantage is to increase the size of the sample. For example, let us consider a sample of size 40 × 40 × 40 mm^3^. First, the effects of such a wide sample are considered because the width of the sample significantly affects the resolution of diffractometry. For example, a difference of *L* between neutrons passing though the left and right ends corresponds to a width of *ΔL* in the case of orthogonal scattering; hence, *ΔL/L* is 0.33% for a sample width of 40 mm when the moderator and detector are located at 10 m and 2 m from the sample, respectively. On the contrary, the ratio of the *L* of the direct beam through the sample centre to that through the sample ends is 0.9999 in transmission spectroscopy. Next, we discuss the influence of thickness, where the powder filling rate is assumed to be the same as that in our experiment using a vanadium cell with a diameter of 10 mm (the average thickness is 8 mm). In this case, the (111) magnetic Bragg edge depth should be 2 × 5 = 10% for the five times thicker NiO sample, while the transmission around the edge is expected to be (*Tra*(*λ* = 0.96 nm))^5^ = (0.25)^5^ = 0.1%. In this case, the total number of neutrons transmitted through the sample of size 40 × 40 × 40 mm^3^ becomes 1.6 × 10^6^ n/s if the incident flux from the MW-class pulsed spallation source is set at 1 × 10^6^ n/s/mm^2^. When the time of flight is divided into 500 parts, each bin contains approximately 10^7^ neutrons after an hour of accumulation. The estimated statistical error of 0.03% is considerably lower than the above mentioned edge depth of 10%. A simple extrapolation for the signal-to-noise ratio suggests that the observation of a magnetic Bragg edge with one hundredth the depth is possible in principle (magnetic moment of 0.19 μ_B_ for the same structure), although sample sizes of 40 mm are generally inconvenient to prepare. Before ending the discussion, we should mention that the detector counts the neutrons arriving late owing to the multiple scattering in such a thick sample of randomly oriented powder. A notable point is that the opening solid angle of the detecting area of 40 × 40 mm^2^ is only 0.003% of the entire solid angle if the detector is located 2 m from the sample. Hence, we can infer that the intensity of multiple scattering is insignificant in comparison to that of the direct beam.

As demonstrated here, the transmission spectroscopy for the magnetic Bragg edges of powders provides evident advantages when studying the magnetic structures at extremely high resolutions with acceptable sensitivity, rather than when studying them under multi-extreme conditions. The above discussion is based on the use of existing MW-class pulsed spallation sources; however, we know that current accelerator technologies can enable us to construct small-scale accelerator-driven neutron facilities^18^. We can measure the magnetic neutron scatterings daily as X-ray diffractions in our laboratory, if the transmission spectroscopy for the magnetic Bragg edges of powders discussed above is utilized in such facilities. The point is that the incident beam generated by small-scale accelerators is extremely weak; the difference in flux is 10^4^–10^5^ times. Based on this, we again note a particular characteristic of the spectroscopy, i.e., beam divergence is unrelated to resolution as long as *L* is constant. The critical reflection angle of the super mirror with 8001 Ni/Ti multilayers is 6.7 times larger than that of natural nickel and is expected to be 7° at a *λ* of 0.96 nm^19^. If we can utilize such mirrors, a neutron within a solid angle of 28° can be principally focused on the sample without decreasing resolution. This solid angle is 10^4^ times larger than that in conventional neutron diffractometry, as an incident beam divergence of 0.3° is a practical limit for a Δ*d*/*d* of 0.5% in an orthogonal scattering measurement^12,17^. This fact indicates new opportunities for determining the approximate spin configuration at the above mentioned resolution/sensitivity in the laboratory, even though further considerations on optical design are required owing to the fact that the moderator is not a point source.

As discussed here, transmission spectroscopy is a promising tool in magnetism. The observation of Bragg dips for single crystals shows a certain potential to advance the frontier of magnetism toward unknown territories under high pressures or high magnetic/electric fields at low temperatures. On the other hand, the measurement of Bragg edges in the powder provides us opportunities to study the spin configuration at extremely high resolution/acceptable sensitivity using MW-class pulsed spallation source facilities and possibly compact neutron sources in laboratories.

## Method

The samples used were a commercially supplied powder and single crystal of nickel oxide; the former was obtained from the Kojundo Chemical Laboratory Co., while the latter was a cuboid 10 mm in height, 10 mm in width, and 4 mm in thickness, manufactured by SurfaceNet GmbH; the crystal orientation was along the (111) plane on the 10 × 10 mm^2^ surface. The powder was sparsely filled in a vanadium cell with a diameter of 10 mm. The single crystal was fixed on the sample holder so that its (111) surface was approximately perpendicular to the incident beam, and it was purposely inclined to the beam at almost 80°. The transmission spectra of the powder and single-crystal samples were measured as functions of the time of flight at beam line 10 (BL10) NOBORU in J-PARC, according to the general use program of MLF (2016A0099). For the perpendicularly fixed crystal, a neutron beam with a diameter of 100 mm and a flux density of 1 × 10^2^ n/s/mm^2^ was irradiated on the (111) surface of the crystal and the transmitted neutrons were counted by a gas electron multiplier (nGEM) detector located 14.5 m away from the neutron source. (Simultaneous measurement of several samples was the reason for using a larger beam size in this work.) For the purposely inclined crystal and powders, a neutron beam with a size of 25 × 20 mm and a flux density of 6 × 10^2^ n/s/mm^2^ was irradiated on the (111) surface of the crystal and the randomly aligned powder. Then, the transmitted neutrons were counted by an Li glass scintillator 14.0 m away from the source.
